# Genetics of obsessive-compulsive disorder

**DOI:** 10.1017/S0033291721001744

**Published:** 2021-10

**Authors:** Behrang Mahjani, Katharina Bey, Julia Boberg, Christie Burton

**Affiliations:** 1 Seaver Autism Center for Research and Treatment, Icahn School of Medicine at Mount Sinai, New York, NY, USA; 2 Division of Tics, Obsessive-Compulsive Disorder (OCD) and Related Disorders, Icahn School of Medicine at Mount Sinai, New York, NY, USA; 3 Department of Psychiatry, Icahn School of Medicine at Mount Sinai, New York, NY, USA; 4 Department of Medical Epidemiology and Biostatistics, Karolinska Institutet, Stockholm, Sweden; 5 Department of Psychiatry and Psychotherapy, University Hospital Bonn, Bonn, Germany; 6 Department of Clinical Neuroscience, Karolinska Institutet, Stockholm, Sweden; 7 Neurosciences and Mental Health, Hospital for Sick Children, Toronto, Canada

**Keywords:** Obsessive-compulsive disorder, obsessive-compulsive symptoms, genetic epidemiology, molecular genetics

## Abstract

**Background:**

Obsessive-compulsive disorder (OCD) is a psychiatric disorder with multiple symptom dimensions (e.g. contamination, symmetry). OCD clusters in families and decades of twin studies clearly demonstrate an important role for genetics in the etiology of the disorder.

**Methods:**

In this review, we summarize the genetic epidemiology and molecular genetic studies of OCD and obsessive-compulsive symptoms.

**Results:**

OCD is a heritable, polygenic disorder with contributions from both common and rare variants, including *de novo* deleterious variations. Multiple studies have provided reliable support for a large additive genetic contribution to liability to OCD, with discrete OCD symptom dimensions having both shared and unique genetic risks. Genome-wide association studies have not produced significant results yet, likely because of small sample sizes, but larger meta-analyses are forthcoming. Both twin and genome-wide studies show that OCD shares genetic risk with its comorbid conditions (e.g. Tourette syndrome and anorexia nervosa).

**Conclusions:**

Despite significant efforts to uncover the genetic basis of OCD, the mechanistic understanding of how genetic and environmental risk factors interact and converge at the molecular level to result in OCD's heterogeneous phenotype is still mostly unknown. Future investigations should increase ancestral genetic diversity, explore age and/or sex differences in genetic risk for OCD and expand the study of pharmacogenetics, gene expression, gene × environment interactions and epigenetic mechanisms for OCD.

## Introduction

Obsessive-compulsive disorder (OCD) is characterized by intrusive obsessions and/or compulsions that are disturbing and time-consuming (American Psychiatric Association, 2013). Obsessions are images, thoughts or urges that are intrusive and unwanted and are associated with anxiety, distress, disgust and/or a sense of something being not-just-right. Common obsessional themes are worries about hurting others, being a bad person or contaminating oneself or others. Compulsions are repetitive behaviors or mental rituals, such as checking for safety, inspecting for cleanliness, repetitive counting or ordering. The function of compulsions is to prevent or reduce anxiety or to impede an (imagined) feared event, evoked by obsessions (Barlow, 2014). For example, an obsessive fear of being contaminated will lead to high levels of distress and compulsive or ritualized washing serves the function of temporarily reducing the distress brought on by the obsession. However, in OCD, the obsessions recur, re-activate the compulsions and the cycle of obsessions and compulsions continues. OCD can also be conceptualized as the extreme of obsessive-compulsive symptoms that are present in the general population (Rachman & de Silva, 1978).

The presentation of OCD is heterogeneous with the content of obsessions and compulsions varying considerably across patients. As a result, patients are sometimes divided into more granular categorical subtypes based on their predominant symptom type or comorbid presentation. For example, patients can be identified as ‘checkers and washers’ (Calamari, Wiegartz, & Janeck, 1999; Khanna, Kaliaperumal, & Channabasavanna, 1990; Khanna & Mukherjee, 1992) or be designated as OCD with and without concurrent tic-disorder (Leckman et al., 1994). Investigators have shown four broad primary symptom dimensions that are not mutually exclusive: (1) contamination and cleaning, (2) symmetry; repeating, ordering and counting (3) forbidden thoughts; sexual, religious, aggressive and (4) hoarding (Bloch, Landeros-Weisenberger, Rosario, Pittenger, & Leckman, 2008; Leckman et al., 1997; Mataix-Cols, do Rosario-Campos, & Leckman, 2005). Symmetry, checking and ordering are often the most common type of symptoms present (Fullana et al., 2010; Vellozo et al., 2021; Williams, Mugno, Franklin, & Faber, 2013); however, the patterns of symptom presentation can vary based on several factors, including age, gender, comorbidities (Vellozo et al., 2021), among others.

The first description of OCD in the psychiatric literature is from 1838 by Jean-Étienne Esquirol and almost a century later, one of the first family studies on OCD was published (Lewis, 1936). Ever since, heredity has been regarded to play an important role in the development of OCD. Until recently, OCD has been classified as an anxiety disorder. This changed in 2013 with Diagnostic and Statistical Manual of Mental Disorders (DSM)-5 when Obsessive-Compulsive and Related Disorders (OCD-RD) was introduced as a new section that included OCD as well as body dysmorphic disorder, skin picking disorder, trichotillomania and hoarding disorder. In International Classification of Diseases (ICD)-11, this classification also includes olfactory reference syndrome and hypochondria. An additional important change with DSM-5 was the removal of the requirement of insight; i.e. the acknowledgment of ‘the irrational nature’ of OCD symptoms, and instead adding a specifier for distinction of fair, poor and absent insight. Current or past tic disorder was also added as a specifier (American Psychiatric Association, 2013).

Treatment for OCD includes pharmacological, psychotherapeutic, and surgical (DBS) options. There is robust evidence that treatment with selective serotonin reuptake inhibitors and cognitive behavioral therapy, including exposure and response prevention therapy, provide relief from OCD symptoms (Skapinakis et al., 2016). Second-line pharmacological options are clomipramine or atypical antipsychotic drugs, mainly risperidone and aripiprazole (Del Casale et al., 2019). However, far from all individuals with OCD respond sufficiently to these interventions (Hirschtritt, Bloch, & Mathews, 2017). With OCD being highly debilitating and prevalent, more efforts are needed in the discovery of underlying mechanisms in order to improve treatment outcome, early detection and understand the etiology of OCD. The overall aim of this review is to give an overview on OCD, the recent progress of genetic and epigenetic research and a perspective on future directions of the field.

## Epidemiology

The lifetime prevalence of OCD is estimated at 1–3%, with largely consistent rates across diverse countries (Fawcett, Power, & Fawcett, 2020; Ruscio, Stein, Chiu, & Kessler, 2010; Weissman et al., 1994). Although females are affected at a slightly higher rate than males in adolescence and adulthood, males are more commonly affected in childhood (Mathes, Morabito, & Schmidt, 2019). Age of onset follows a bimodal distribution, peaking at adolescence (13–18 years) and early adulthood (Albert et al., 2015; Anholt et al., 2014). This bimodal pattern suggests that childhood-onset OCD represents a distinct subtype of the disorder with specific clinical features and potentially different etiological factors (Geller et al., 1998). In fact, early-onset OCD has been associated with a stronger genetic component (Nicolini, Arnold, Nestadt, Lanzagorta, & Kennedy, 2009), a higher comorbidity with tic-related disorders (which often have overlapping clinical symptoms with OCD), and more severe symptom severity than late-onset OCD (Stewart et al., 2004; Taylor, 2011).

OCD can be a severely impairing disorder and have significant negative effects on various aspects of quality of life, especially affecting social relationships (Stein et al., 2019; Subramaniam, Soh, Vaingankar, Picco, & Chong, 2013). Patients with OCD have a significantly increased risk of death by natural or unnatural causes as compared to the general population (mortality risk ratio = 1.68 and 2.61, respectively; Meier et al., 2016). Similarly, a recent review reported substantial proportions of OCD patients experiencing suicidal ideation, and a mean rate of lifetime suicide attempts of 14.2% (Albert, De Ronchi, Maina, & Pompili, 2019; Fernández de la Cruz et al., 2017).

The majority of OCD patients exhibit at least one comorbid disorder, with major depressive disorder, obsessive-compulsive personality disorder, generalized anxiety disorder, specific phobia and social anxiety disorder being the most common co-occurring diagnoses. Other common comorbidities include autism spectrum disorder (ASD), anorexia nervosa and attention-deficit hyperactivity disorder (ADHD) (Brakoulias et al., 2017). Major depression is also the most prevalent lifetime comorbidity of OCD, with a prevalence of 50% across various countries (Brakoulias et al., 2017). Furthermore, OCD can be associated with Tourette syndrome or neurologic conditions, including stroke, traumatic brain injury, progressive supranuclear palsy, Huntington disease, Parkinson's disease and various dementias (Drubach, 2015; Richter & Ramos, 2018).

Multiple risk factors may contribute to the development of OCD, including both genetic and environmental risk factors, such as perinatal complications, childhood trauma, reproductive cycle events (e.g. age of onset of menarche) and stressful life events (as reviewed in detail elsewhere; Brander, Pérez-Vigil, Larsson, & Mataix-Cols, 2016; Raposo-Lima & Morgado, 2020). However, the majority of the studies assessing environmental risk factors are based on retrospective self-reports, limiting causal inference. A persistent low-grade inflammation involving both innate and adaptive immune systems has also been observed in OCD (Gerentes, Pelissolo, Rajagopal, Tamouza, & Hamdani, 2019). Most prominently, it has been proposed that an autoimmune response to infection leading to inflammation in the basal ganglia may underlie some cases of unusually abrupt early-onset OCD (Swedo et al., 1998). This condition was initially termed *pediatric autoimmune neuropsychiatric disorders associated with streptococcal infections* (PANDAS), but has recently been broadened to *pediatric autoimmune neuropsychiatric syndrome* (PANS), emphasizing that the illness may start with infectious triggers other than streptococcal (Chiarello, Spitoni, Hollander, Matucci Cerinic, & Pallanti, 2017). Notably, OCD itself represents a risk factor for the subsequent diagnosis of other psychiatric disorders, such as schizophrenia (Cheng et al., 2019; Meier et al., 2014) and anorexia nervosa (Cederlöf et al., 2015), suggesting an etiological overlap.

With regard to the neurophysiological underpinnings of OCD, mounting evidence from neuroimaging, neuropsychological and pharmacological studies suggests a dysfunction in the cortico-striato-thalamo-cortical (CSTC) circuitry (Richter & Ramos, 2018). The CSTC (or frontostriatal) model of OCD postulates an imbalance between direct and indirect pathways from cortical brain regions, such as the orbitofrontal cortex (OFC) and the anterior cingulate cortex (ACC), to the thalamus via the striatum, leading to a reduced inhibition of the thalamus and thus an increased excitatory feedback to frontal brain regions (Pauls, Abramovitch, Rauch, & Geller, 2014). The resulting hyperactivity in the OFC has been linked to repetitive thoughts and persistent concerns about harm, i.e. obsessions. In order to neutralize the distress resulting from the perceived threat, OCD patients perform compulsions, whose repetitive and ritualistic nature is supported by the striatum (Saxena & Rauch, 2000). Recent findings from the ENIGMA Consortium also highlight cortical and subcortical abnormalities in OCD (van den Heuvel et al., 2020). For example, adult OCD patients exhibited smaller hippocampal and larger pallidal volumes compared to controls, whereas unmedicated pediatric patients with OCD had a larger volume of the thalamus than controls (Boedhoe et al., 2017). Furthermore, both adult and pediatric OCD were associated with a thinner inferior parietal cortex (Boedhoe et al., 2018).

## Genetic epidemiology

### Familial clustering of OCD

Consistent evidence demonstrates that OCD clusters in families. As summarized by Browne, Gair, Scharf, and Grice (2014), the estimated recurrence risk among first-degree relatives for lifetime OCD is between 6% and 55% (Bienvenu et al., 2012; do Rosario-Campos et al., 2005; Fyer et al., 2005; Grabe et al., 2006; Hanna, Himle, Curtis, & Gillespie, 2005; Nestadt et al., 2000; Pauls, Alsobrook, Goodman, Rasmussen, & Leckman, 1995). In comparison, the lifetime prevalence of OCD in the general population is estimated at 1–3% (Fontenelle, Mendlowicz, & Versiani, 2006; Ruscio et al., 2010). A few studies have demonstrated that the prevalence of OCD is substantially higher in the relatives of probands with early onset (i.e. ⩽18 years old) compared to later onset OCD (Arumugham et al., 2014; Nestadt et al., 2000; Pauls et al., 1995). For example, in the Hopkins OCD Family Study, the prevalence of OCD in relatives of probands with an early onset was 13.8%, compared to 0% in probands with later onset (*p* = 0.006) (Nestadt et al., 2000).

Familial clustering of OCD has also been investigated for obsessive-compulsive symptom dimensions. Studies have demonstrated that familial recurrence risk estimates are even higher among the family members of probands for obsessive-compulsive symptoms and behaviors (do Rosario-Campos et al., 2005; Fyer et al., 2005; Grabe et al., 2006; Pauls et al., 1995). The familiality of symptom dimension types appears to vary. Hoarding and contamination/cleaning symptoms are reported to have the highest familial risk (Brakoulias et al., 2016) whereas symmetry-related symptoms may be more prevalent in familial OCD than sporadic OCD (Viswanath, Narayanaswamy, Cherian, Reddy, & Math, 2011).

Analyses of relatives of individuals with OCD and obsessive-compulsive symptoms strongly support a significant genetic contribution and shared risk factors to the liability of OCD and sub-diagnostic OCD symptoms. In addition, a comparison of recurrence risk among different family types showed patterns close to that expected under an additive genetic model (Mahjani et al., 2020; Mataix-Cols et al., 2013).

Multiple studies have reported that OCD, Tourette syndrome (Hirschtritt et al., 2018; Mathews & Grados, 2011; Pinto et al., 2016; Zilhão, Smit, Boomsma, & Cath, 2016), ADHD (Abramovitch, Dar, Mittelman, & Wilhelm, 2015; Geller et al., 2007; Mathews & Grados, 2011), anxiety disorders (López-Solà et al., 2014) and ASDs (Meier et al., 2015) overlap in their genetic phenomenological features. Browne et al. showed that OCD and Tourette syndrome cluster in families (Browne et al., 2015). The familial aggregation of Tourette syndrome was substantially higher than the familial aggregation for OCD. In addition, they observed a significant cross-disorder recurrence risk. OCD, Tourette syndrome and ADHD co-occur in clinical and epidemiological samples (Mathews & Grados, 2011; Pinto et al., 2016). Family studies have reported evidence of shared familial transmission between these disorders (Pinto et al., 2016). The co-occurrence can be partly explained by shared etiological influences (Pinto et al., 2016). Further research examining sub-dimensions of these phenotypes is warranted.

### Heritability from family and twin studies

**Fig. 1.**
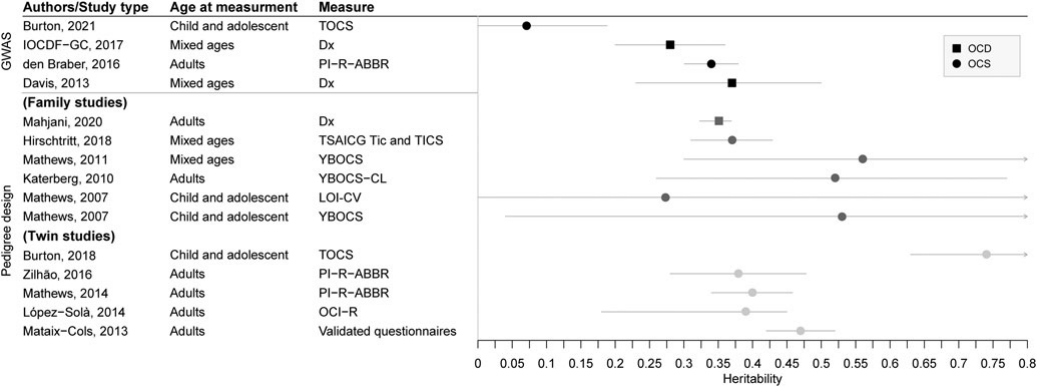
Heritability of OCD and obsessive-compulsive symptoms (OCS). OCD, obsessive-compulsive disorder; OCS, obsessive-compulsive symptoms; TOCS, Toronto Obsessive-Compulsive Scale; Dx, diagnosis by a clinician; PI-R-ABBR, Padua Inventory Revised Abbreviated; TSAICG, Tic and Comorbid Symptom (TICS) Inventory; YBOCS, Yale-Brown Obsessive-Compulsive; YBOCS-CL, Yale-Brown Obsessive Compulsive Scale-Checklist; LOI, Leyton Obsessional Inventory. We only included studies that reported standard error or confidence intervals for the estimate of heritability. If multiple studies used the same data, we included the first study.

Twin and family studies have been reviewed previously in detail by Purty et al. and Browne et al. (Browne et al., 2014; Viswanath, Purty, Nestadt, & Samuels, 2019). Table 1 and Fig. 1 summarize the estimation of heritability of OCD and obsessive-compulsive symptoms using different study types and assessment methods. Large twin studies of OCD consistently reported that the monozygotic (MZ) twin correlation is more than twice as high as dizygotic (DZ) twin correlation (Mataix-Cols et al., 2013; Monzani, Rijsdijk, Harris, & Mataix-Cols, 2014). Similar patterns were observed for obsessive-compulsive symptoms with a reported concordance rate of 87% in 15 MZ twin pairs and 47% in DZ twin pairs (Carey & Gottesman, 1981). Twin studies consistently show the substantial heritability of OCD and obsessive-compulsive symptoms (Table 1). Monzani et al. reported 48% as the overall heritability for OCD in one of the most statistically robust twin studies (Monzani et al., 2014), and Mataix-Cols et al. showed that familial risk for OCD was largely attributable to additive genetic factors (47%), with no significant effect of shared environment (Mataix-Cols et al., 2013). In a childhood-onset sample, the estimated heritability was 45–61% for obsessive-compulsive symptoms using 4246 twin pairs (Hudziak et al., 2004). The heritability of obsessive-compulsive symptoms is slightly lower in adults (30–40%) than in children (45–58% for 12-year-old twins and 55% for 6-year-old twins; Bolton et al., 2009; Hudziak et al., 2004; Zilhão et al., 2015). In both adults and children, each OCD symptom dimension is heritable and co-heritable, although each dimension also has some genetic variance that is unique to them as well, particularly hoarding (Burton et al., 2018; Iervolino, Rijsdijk, Cherkas, Fullana, & Mataix-Cols, 2011; Mathews et al., 2014; van Grootheest et al., 2008a). Multiple studies have reported genetic correlations between OCD, tic disorders (Pinto et al., 2016; Zilhão et al., 2016), anorexia nervosa (Cederlöf et al., 2015) and ADHD (Pinto et al., 2016) using twin and family studies. As an example, population-based twin studies have reported a genetic correlation of 0.35 between hoarding and tics, and 0.37 between obsessive-compulsive symptoms and tics (Zilhão et al., 2016). Few studies have examined sex differences in the estimate of OCD heritability, and results are mixed. Hur et al. reported a higher heritability estimate in males than in females (53% *v.* 41%) for the Maudsley Obsessional-Compulsive Inventory (Hur & Jeong, 2008), whereas Grootheest et al. reported no genetic sex differences in OCI-R (van Grootheest et al., 2008a). It should be noted that the estimates of heritability from twin studies can be inflated since the amount of variance attributable to the common environment may not be identical in MZ and DZ pairs (Felson, 2014). Mahjani et al. showed that assortative mating (Peyrot, Robinson, Penninx, & Wray, 2016) and maternal effects (genetic nurture effects; Wolf & Wade, 2009) can also inflate the estimate of heritability (Mahjani et al., 2020).

**Table 1.** Heritability estimates (twin studies, family studies and GWAS)

Authors, Year	Assessment method	Size	Age	Heritability estimate
Twin studies				
Torgersen (1980)	Oral, obsessive, and hysterical personality syndromes	99 twin pairs	Mean: 41	18% (male) 23% (female)
Clifford, Murray, and Fulker (1984)	Leyton Obsessional Inventory	419 twin pairs	Mean: 31	47%
Jonnal, Gardner, Prescott, and Kendler (2000)	Padua Inventory of Obsessive-Compulsiveness	527 twin pairs	Mean: 36	33% (compulsiveness) 26% (obsessiveness)
Eley et al. (2003)	16-item questionnaire	4564 twin pairs	Age 4	54%
Hudziak et al. (2004)	CBCL	4246 twin pairs	Age 7, 10, 12	45–58%
Van Grootheest, Cath, Beekman, and Boomsma (2007)	YASR-OCS	5893 twins and 1304 additional non-twin siblings	Mean: 22.4	47% (39% for men and 50% for women)
van Grootheest et al. (2008a)	Youth Self-Report Obsessive-Compulsive Scale	Age 12, 746 twin pairs; age 14, 963 twin pairs; age 16 1070, twin pairs	Age 12, 14 and 16	7% at the age of 12, 57% at the age of 14, 54% at the age of 16
van Grootheest, Boomsma, Hettema, and Kendler (2008b)	Twenty items from PI	1383 female twin pairs	Mean: 36.6	36%
Hur and Jeong (2008)	MOCI	751 twin pairs	Range: 13–23	53% male, 41% female
Iervolino et al. (2009)	HRS-SR	5022 twin pairs	Mean: 55.5	50% (hoarding)
van Grootheest, Cath, Hottenga, Beekman, and Boomsma (2009)	PI-R-ABBR	Twins from 4198 families	Mean: 17.8, 19.8, 25.5, 33 (4 time points)	Around 40% at each time-point
Iervolino et al. (2011)	OCI-R	2053 twin pairs	Range: 17–86	38–47%
Mataix-Cols et al. (2013)	Validated questionnaire	16 383 twin pairs	Range: 20–7 (for twins)	47% (for twins)
Zilhão et al. (2015)	PI-ABBR	10 134 in 2002 and 15 720 in 2008 (twin pairs and their siblings)	Mean: 33	56% (longitudinal broad sense heritability)
Monzani et al. (2014)	OCI-R	5409 twin pairs	Range: 16–90	48%
López-Solà et al. (2014)	OCI-R	2495 twin pairs	Range: 18–45	42% (male) 38% (female)
Mathews et al. (2014)	PI-ABBR	Total sample = 15 914, 7906 twin pairs	Mean: 41.3	40%
Pinto et al. (2016)	Validated questionnaire	21 911 twin pairs	Mean: 33	45%
Zilhão et al. (2016)	PI-ABBR	5293 twin pairs	Mean: 33.61	38%
Burton et al. (2018)	TOCS	220 twin pairs	Mean: 11	74%
Family studies				
Mathews et al. (2007)	YBOCS and LOI-CV	11 multigenerational families with OCD and hoarding (*N* = 92; 44 with OCD)	Mean: 37.2	53.06% (YBOCS−CV) 27.28% (LOI)
Katerberg et al. (2010)	YBOCS-CL	52 OCD-affected multigenerational families (1224 OCD subjects)	Mean: 33.8	52%
Mathews and Grados (2011)	YBOCS	952 individuals from sib-pairs	Range: 4–77	56%
Hirschtritt et al. (2018)	TSAICG tic and comorbid symptom inventory	3494 individuals from 1365 families	Over 6	37%
Mahjani et al. (2020)	Clinical diagnosis, ICD-10	Population cohort of 822 843 individuals, 7184 diagnosed with OCD	Range: 23–31	35% (7.6% genetic maternal effects)
Genome-wide association studies				
Davis et al. (2013)	Clinical diagnosis	1061 OCD cases and 4236 OCD controls	Over 18	37%
Den Braber et al. (2016)	PI-R-ABBR	6881 individuals from twin and sib pairs	Mean: 46.7	34%
Guo et al. (2017)	DSM-IV criteria for OCD	2998 individualsin nuclear families	Mean: 9.4	42.7%
International Obsessive Compulsive Disorder Foundation Genetics Collaborative (IOCDF-GC) and OCD Collaborative Genetics Association Studies (OCGAS) (2018)	Clinical diagnosis	2688 OCD cases and 7037 controls		28%
Yilmaz et al. (2018)	Clinical diagnosis	2688 OCD cases, and 18 013 controls		29%
Khramtsova et al. (2019)	Clinical diagnosis	Data from: Stewart et al. (2013); Mattheisen et al. (2014); International Obsessive Compulsive Disorder Foundation Genetics Collaborative (IOCDF-GC) and OCD Collaborative Genetics Association Studies (OCGAS), 2018	Over 18	13.1% (male), 29.6% (females) (the difference was not statistically significant) 22.5% (combined)
Burton et al. (2021)	TOCS	5018 unrelated Caucasian children and adolescents	Range: 6–18	7.1%

CBCL, Child Behavior Checklist; YASR-OCS, Young Adult Self Report Obsessive-Compulsive Scale; PI, Padua Inventory; MOCI, Maudsley Obsessional-Compulsive Inventory; HRS-SR, Hoarding Rating Scale-Self-Report; PI-R-ABBR, Padua Inventory Abbreviated Revised; OCI-R, Obsessive-Compulsive Inventory-Revised; PI-ABBR, Padua Inventory Abbreviated Revised; TOCS, Toronto Obsessive-Compulsive Scale; YBOCS, Yale-Brown Obsessive-Compulsive Scale; LOI-CV, Leyton Obsessional Inventory, Childhood Version; YBOCS-CL, Yale-Brown Obsessive Compulsive Scale-Checklist.

Studies in this table are based on (1) PubMed search on 2/18/2021 using keywords ‘(obsessive[title] OR compulsive[title] OR obsessions[title] OR compulsions[title] OR OCD[title]) AND heritability’; (2) book chapter by Grünblatt (2021) and (3) review article by Browne et al. (2014).

## Molecular genetics

### Common variants

Initial molecular genetic studies of OCD focused on candidate genes related to neurochemical systems associated with the disorder (for a review, see, Pauls et al., 2014). As with all other psychiatric disorders, results from these studies have been very mixed with few replicated findings (Pauls et al., 2014). Genome-wide studies of OCD are still in their infancy. The first GWAS included 1465 patients with OCD with mixed ages of symptom onset, 5557 controls and 400 trios from the International Obsessive-Compulsive Consortium (Stewart et al., 2013). There were no genome-wide significant variants identified although a locus near BTB domain containing 3 (*BTBD3*) reached genome-wide significance (*p* = 3.84 × 10^−8^) in the trio-subset. The second GWAS, which included 5061 individuals (1065 families with 1406 patients with OCD with onset in childhood and adolescence and population-based controls) from the OCD Collaborative Genetics Association Study (OCGAS) consortium, also reported no genome-wide significant loci (Mattheisen et al., 2014). The locus with the smallest *p* value was upstream of protein tyrosine phosphatase receptor type D (*PTPRD*, *p* = 4.13 × 10^−7^).

Most recently, these two GWAS have been meta-analyzed with a total sample of 2688 patients with OCD of European descent and 7037 genomically matched controls [International Obsessive Compulsive Disorder Foundation Genetics Collaborative (IOCDF-GC) and OCD Collaborative Genetics Association Studies (OCGAS), 2018]. As this sample was still underpowered, no genome-wide loci were identified. The loci with the smallest *p* values were in or close to haplotype blocks or genes including cancer susceptibility 8 (*CASC8/CASC11*), glutamate ionotropic receptor delta type subunit 2 (*GRID2*), KIT proto-oncogene receptor tyrosine kinase (*KIT*), ankyrin repeat and SOCS box containing 13 (*ASB13*), *GRIK2*, *CHD20*, *DLGAP1*, fas apoptotic inhibitory molecule 2 (*FAIM2*), *PTPRD* and R-spondin 4 (*RSPO4*). Several of these genes were identified in the previous studies (Mattheisen et al., 2014; Stewart et al., 2013) and tag neurochemical systems previously associated with OCD including glutamate (Pauls et al., 2014). The Psychiatric Genomics Consortium (PGC) is currently working on a substantially larger meta-analysis that will include at least 14 000 patients with OCD and over 560 000 controls.

In addition to GWAS of OCD diagnosis, there have been three genome-wide analyses focusing on obsessive-compulsive symptoms or traits in larger population-based samples (Burton et al., 2021; Den Braber et al., 2016; Smit et al., 2019). The first included 6931 samples from the Netherlands Twin Registry (NTR) study that measured obsessive-compulsive symptoms using the Padua Inventory-Revised (Den Braber et al., 2016). The study identified a genome-wide significant locus in BLOC-1-related complex subunit 8 (*BORCS8* or *MEF2BNB*) gene (*p* = 2.56 × 10^−8^) whereas a gene-based analysis similarly reported significant associations with genes in the myocyte enhancer factor 2B (*MEF2B*) family. A recent GWAS in a sample of children and youth (*n* = 5018) identified a genome-wide significant locus in *PTPRD* (Burton et al., 2021).

These GWAS of obsessive-compulsive symptoms are beginning to shed light on the genomics of OCD. The locus in *PTPRD* was also associated with OCD case/control status in a meta-analysis of independent samples (Burton et al., 2021). Polygenic risk scores (PRSs) for obsessive-compulsive symptoms or traits are also associated with diagnosed OCD and/or vice versa (Burton et al., 2021; Den Braber et al., 2016). Currently, genetic correlations between obsessive-compulsive symptoms and OCD case/control are moderate but non-significant (*r*_G_ = 0.42–0.83, *p* > 0.07), likely because of insufficient sample sizes (Burton et al., 2021; Smit et al., 2019). Together, these results suggest that obsessive-compulsive symptoms do share genetic risk with OCD, although this sharing may depend on the types of symptoms. Additionally, these findings support the hypothesis that OCD may reflect the extreme of obsessive-compulsive symptoms that are distributed in the population, as observed in other disorders (Thapar & Cooper, 2016). Larger samples will be necessary to understand to what extent obsessive-compulsive symptoms and OCD share genetic risks.

The heterogeneous nature of OCD is reflected in differences in genetic results across symptom dimensions. Focusing on compulsive symptoms only also increased the ability to identify genome-wide significant genes. Recently, a smaller study of 399 patients with OCD using a gene-based analysis has reported that *SETD3* was associated with hoarding symptoms only (*p* = 1.89 × 10^−8^) and that biological pathways and processes were differentially associated across symptom dimensions (Alemany-Navarro et al., 2020).

### Genetic architecture

Single-nucleotide polymorphism (SNP)-based heritability from GWAS studies has also shed light on the genetic architecture of OCD. As shown in Fig. 1, the estimates of SNP heritability can vary based on measurement and ascertainment. Heritability of OCD from common SNPs [minor allele frequency of ⩾1–5%) ranges from 0.25 to 0.43 depending on the age of onset and ascertainment (Davis et al., 2013; International Obsessive Compulsive Disorder Foundation Genetics Collaborative (IOCDF-GC) and OCD Collaborative Genetics Association Studies (OCGAS), 2018; Mattheisen et al., 2014]. Interestingly, population-based studies report lower SNP heritability based on common SNPs for obsessive-compulsive symptoms (0.06–0.14; Burton et al., 2021; Den Braber et al., 2016; Smit et al., 2019) than for clinical OCD [Davis et al., 2013; International Obsessive Compulsive Disorder Foundation Genetics Collaborative (IOCDF-GC) and OCD Collaborative Genetics Association Studies (OCGAS), 2018; Mattheisen et al., 2014]. The reason for this difference is currently unclear but has been observed for other disorders including ADHD (Demontis et al., 2019; Middeldorp et al., 2016). SNP heritability also differs based on general OCD symptom type. Compulsive symptoms rather than obsessive symptoms had higher SNP heritability and genetic correlations with OCD (Smit et al., 2019). Heritability for SNPs with a minor allele frequency between 0.1% and 5% for OCD is reported to be 0% (Davis et al., 2013); however, these results are not yet replicated (Mahjani et al., 2021). Given that twin- and SNP-based heritability estimates are in a similar range, particularly for adult OCD (van Grootheest, Cath, Beekman, & Boomsma, 2005), this suggests that common genetic variants account for a substantial portion of the variance for OCD (Davis et al., 2013).

### Rare variants

Rare variants have also been implicated in OCD. Older studies identified cytogenetic abnormalities present in patients with OCD (Fernandez, Leckman, & Pittenger, 2018). More recent high-resolution studies also implicate rare copy number variants (CNVs) and single-nucleotide variants. Genome-wide scans have revealed that the overall rate of CNV burden does not differ between OCD patients and controls, although potentially for larger CNVs, there is an enrichment of genes related to the brain as well as an increased burden of neurodevelopmental CNVs (Gazzellone et al., 2016; Grünblatt et al., 2017; McGrath et al., 2014). Specific rare CNVs found in OCD patients are in genes or loci (e.g. *PTPRD*, *BTBD9*, *NRXN1*, *ANKS1B*, 16p13.11) previously linked to OCD, Tourette syndrome and neurodevelopmental disorders (Gazzellone et al., 2016; Grünblatt et al., 2017; McGrath et al., 2014). A rare small microdeletion encompassing the *FMN1* gene was identified in a genome-wide scan of early onset OCD patients (*n* = 16; Cappi et al., 2014). Next-generation sequencing in OCD is still in its infancy. Family-based whole exome sequencing of 184 trios with an affected OCD proband and 777 trios with unaffected probands suggests that rare *de novo* variants contribute risk to OCD in 22% of cases (Cappi et al., 2020). This study also identified two novel risk genes for OCD (*CHD8*, *SCUBE1*) based on damaging *de novo* variants and found that *de novo* variants in OCD were enriched in genes previously associated with neurodevelopmental disorders, similar to previous genome-wide CNV studies. Targeted sequencing of evolutionarily constrained regions identified a significant association (*p* = 6.37 × 10^−11^) of pooled coding variants in *NRXN1* in 592 OCD patients compared to 33 370 population controls (Noh et al., 2017). Overall, evidence to date implicates rare variants in the pathogenesis of OCD but future larger genome-wide studies will be critical in improving our understanding.

### Epigenetics and gene expression

Large-scale epigenetic studies are currently lacking for OCD and have focused almost exclusively on DNA methylation. An epigenome-wide scan of DNA methylation from blood reported several genes previously associated with OCD to be differentially methylated between OCD patients (*n* = 65) and controls (Yue et al., 2016). Other small studies using neonatal blood spot and saliva samples have not identified any significant differences between cases and controls; however, DNA methylation did vary with OCD severity and symptoms (Goodman et al., 2020; Nissen et al., 2016). Targeted candidate gene studies of *OXTR* and the serotonin transporter gene (*SLC6A4*) suggest that age of symptom onset and tissue type may play a role (Cappi et al., 2016; Grünblatt et al., 2018; Park, Kim, Jeon, Kang, & Kim, 2020).

Only one study to date has examined genome-wide gene expression in OCD (Song, Liu, Wu, Zhang, & Wang, 2018). Expression of 51 mRNAs from whole blood were significantly different between 30 patients with OCD and 30 paired healthy controls, with the most expressed pathway in these mRNAs being ribosomal. A GWAS study of obsessive-compulsive symptoms examining annotated eQTLs from gene expression data reported an enrichment of genes expressed in the brain, particularly the ACC, nucleus accumbens, amygdala (Smit et al., 2019). As larger samples become available, our understanding of gene expression in OCD will improve.

### Genetic relationships with comorbid disorders

OCD co-occurs with several psychiatric disorders to varying degrees including anxiety disorders, ASD, tics/Tourette syndrome, anorexia nervosa, ADHD, among others (Brakoulias et al., 2017). These phenotypic relationships may be driven in part by shared genetic risk. Recent large-scale cross-disorder papers focused on common variants have revealed significant shared common genetic risk between OCD with AN (*r*_G_ = 0.50 ± 0.12) and Tourette syndrome in particular (*r*_G_ = 0.41 ± −0.10) but also bipolar disorder (*r*_G_ = 0.31 ± 0.07) and schizophrenia (*r*_G_ = 0.35 ± 0.06) (Brainstorm Consortium et al., 2018; Cross-Disorder Group of the Psychiatric Genomics Consortium, 2019; Yilmaz et al., 2018; Yu et al., 2015). ASD and OCD have some common phenotypes (e.g. repetitive behaviors) but do not appear to be genetically correlated (*r*_G_ = 0.001, s.e. = −0.11, ns) (Brainstorm Consortium et al., 2018; Cross-Disorder Group of the Psychiatric Genomics Consortium, 2019). Although OCD and ADHD are somewhat comorbid, these disorders appear to have few shared genetic common risk factors (*r*_G_ = −0.07, s.e. = −0.1, ns) (Brainstorm Consortium et al., 2018; Ritter et al., 2017). Three recent gSEM studies found that OCD clustered with anorexia nervosa consistently but these disorders clustered into either a compulsive behavior cluster with Tourette syndrome (Cross-Disorder Group of the Psychiatric Genomics Consortium, 2019; Grotzinger et al., 2020) or a thought problems cluster with bipolar and schizophrenia (Waldman, Poore, Luningham, & Yang, 2020). This difference in broader clustering is not surprising given that the disorders included in these studies were not identical (e.g. Tourette syndrome and anxiety were not in all studies). However, the relationship between OCD and anorexia nervosa is a consistent theme and as sample sizes increase, gSEM will be an important mechanism to identify possible shared biological mechanisms between disorders and inform nosology.

### Sex differences

Sex differences observed in symptom onset and presentation may be related to underlying genetic differences. Only one study to date has examined sex differences in genetic architecture of OCD (Khramtsova et al., 2019) that used data from the most recent OCD meta-analysis [International Obsessive Compulsive Disorder Foundation Genetics Collaborative (IOCDF-GC) and OCD Collaborative Genetics Association Studies (OCGAS), 2018]. OCD in males and females was highly correlated (*r*_G_ = 1.04, *p* = 0.001) and there was no evidence for a sex-dependent liability model. However, two genes associated with OCD in females (*GRID2*, *GRP135*) were not associated with OCD in males. Future studies with larger samples will be key to further elucidate the role of genetics in sex differences in OCD.

## Clinical and therapeutic implications

Genetic studies aim to identify specific genetic risk factors and biological underpinnings of OCD in order to identify inroads for prevention and to discover or improve existing treatments. Although we have made progress in our understanding of the genetic architecture of OCD, our understanding of the genetics/genomics of OCD remains incomplete. As larger samples become available, we will be able to identify replicated genetic risk factors for OCD and calculate PRS for OCD that will hopefully be helpful in identifying individuals at risk or likelihood of responding to certain treatments.

Pharmacogenetic studies have focused on the role of genetic factors in variable treatment responses (Zai, Brandl, Müller, Richter, & Kennedy, 2014). However, since the candidate gene approach has dominated the field until recently, results should be assessed with that in mind. Several studies have examined associations between drug response in OCD patients and candidate genes, including (1) pharmacokinetic regulating genes, such as *CYP2D6* and *CYP2C19*; (2) serotonergic genes, such as *SLC6A4* and *HTR2A*; (3) glutamatergic genes, such as *SLC1A1* and *DLGAP2*; (4) dopaminergic genes, such as *COMT* and *DRD2* and (5) others, such as *BDNF* and *NTRK3* (see Fineberg et al., 2020). Most notably, a recent GWAS identified a genome-wide significant locus in *DISP1* associated with treatment response to SRIs (Qin et al., 2015). However, no consensus with sufficiently robust evidence exists in the pharmacogenetics of OCD, as many studies did not employ double-blind crossover designs, used a variety of drugs and doses as well as various cutoffs and measures determining response (Fineberg et al., 2020). Future studies examining predictors of treatment response may shift the focus from candidate genes to PRS, in order to inform clinical decision-making toward more personalized therapeutic interventions (for a review, see Murray et al., 2021).

Based on findings from genetic studies, other drugs for OCD have been proposed. Specifically, the implication of glutamatergic genes in the risk for OCD and the role of glutamate as the primary neurotransmitter within the CSTC circuitry have inspired glutamate-modulating drugs such as memantine as potential treatment options for OCD (Pauls et al., 2014). A recent meta-analysis found that memantine showed positive effects as an augmentation therapy in OCD (Modarresi, Chaibakhsh, Koulaeinejad, & Koupaei, 2019). Moreover, glutamatergic anticonvulsant drugs (lamotrigine and topiramate) and riluzole may provide therapeutic benefits to some OCD patients (Marinova, Chuang, & Fineberg, 2017). Ketamine, an *N*-methyl-d-aspartate receptor antagonist, may also be of interest due to its potential for a rapid onset of action, but further randomized placebo-controlled trials in larger study populations are necessary to draw definitive conclusions.

Although too soon for specific clinical implications, recent gSEM studies have also begun to challenge the nosology of psychiatric disorders including OCD. For example, although OCD is commonly thought of as an anxiety disorder (Tynes, White, & Steketee, 1990), genetically it appears to cluster more closely with anorexia nervosa and Tourette syndrome, which may be more compulsive in nature. Although current sample sizes are insufficient to draw firm conclusions, these types of analyses with well-powered samples may continue to shape our understanding of how disorders should be classified and what drives comorbidity and similar traits across disorders (Lee, Feng, & Smoller, 2021).

## Conclusions and future directions

**Fig. 2.**
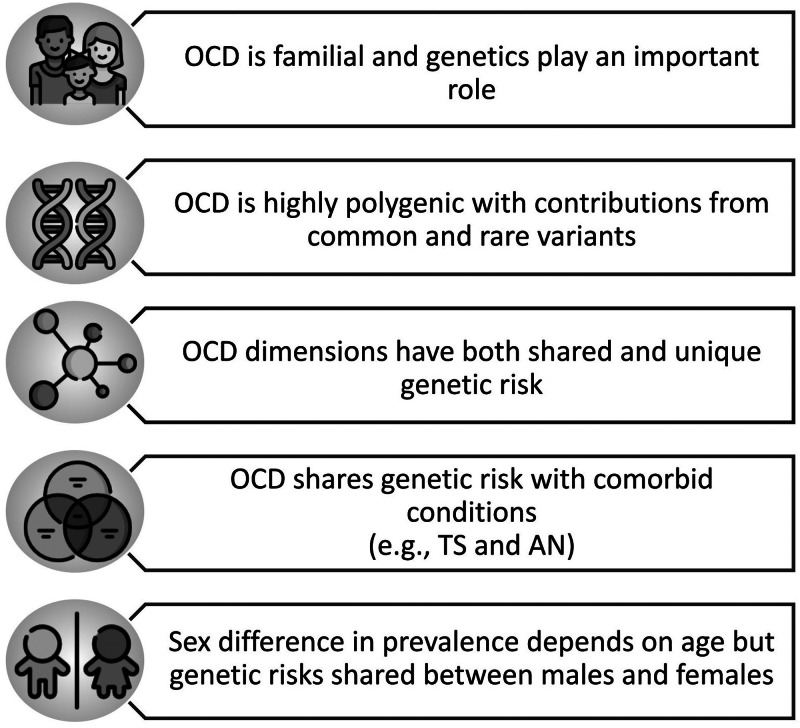
General themes from OCD genetic studies. This figure focuses on findings from genetic epidemiology family-based studies and more recent large-scale molecular genetic studies.

Overall conclusions are presented in Fig. 2. OCD is a heritable, polygenic disorder with contributions from both common and rare variants, in addition to environmental risk factors. Multiple studies have provided reliable evidence for a large additive genetic contribution to liability for OCD and obsessive-compulsive symptoms. To date, no genome-wide significant loci have been identified for OCD, likely due to inadequate sample sizes. These relatively smaller sample sizes also must be considered in the interpretation of results from genetic correlation, polygenic risk and gSEM studies to date as many of these studies are underpowered. However, the PGC is currently working on a substantially larger meta-analysis with encouraging preliminary results. These larger samples will be critical to identify robust genetic variants for OCD and understand its genetic architecture and genetic relationships with other mental health disorders and traits.

Although existing studies have yielded important basic findings, a detailed characterization of the genetic underpinnings of OCD will depend on larger samples and exploring alternative phenotyping strategies beyond case/control status. Measuring obsessive-compulsive symptoms more broadly has produced some promising results to date and this complementary approach will likely improve our understanding of OCD but also comorbid conditions with compulsive-type symptoms. Although one approach may be to narrow down OCD's heterogeneous phenotype by focusing on specific symptom dimensions, another approach may be to broaden the phenotype by examining OCD-RD. Results from twin and family studies as well as GWAS show that OCD shares a substantial genetic overlap with Tourette syndrome and anorexia nervosa (Brainstorm Consortium et al., 2018), and transdiagnostic analyses may facilitate the identification of risk genes by increasing sample size significantly. On the contrary, it will also be important to examine how OCD is genetically distinct from related disorders, such as hoarding, and to characterize the genetic relationship with common comorbidities. Moreover, OCD is relevant to the Research Domain Criteria perspective (Cuthbert & Insel, 2013), which aims to define the contribution of transdiagnostic dimensions such as compulsivity rather than focusing on distinct diagnostic categories (Robbins, Vaghi, & Banca, 2019).

There are several important avenues for future research. So far, the majority of GWAS samples have come from cohorts of European ancestry. The addition of non-European ancestry samples is critical for improving our understanding of the genetics and biology of OCD and improving prediction accuracy but will also help ensure equity in outcomes and possible clinical insights (Bentley, Callier, & Rotimi, 2017; Peterson et al., 2019). The role of environmental factors and how they interact with genetic factors will be important to explore, particularly when considering genetically diverse participants. It will also be important to understand if the genetic architecture and risk for OCD differ based on age of onset (child *v.* adult) as this may inform nosology and risk prediction. Sex differences also require further investigation. Although twin- and family-based studies as well as GWAS suggest that there is likely overlap in genetic risk for OCD between sexes, it is still unclear how this varies by age and symptom dimension. There is also no consensus in the pharmacogenetics of OCD and to date the majority of studies have been candidate gene focused. Although currently limited by sample sizes, future PRS analyses are likely to play an important role in multivariate models with other risk factors to predict diagnosis, symptom severity, disease trajectories, comorbidities and treatment response (Fullerton & Nurnberger, 2019; Wray et al., 2021). Accurate risk prediction will be critical for improving prevention, treatment and outcomes. To strengthen causal inference regarding pathways of potential risk genes, Mendelian randomization should be employed. Furthermore, future studies should examine the role of environmental factors in terms of gene × environment interactions, other structural variants such as variable number of tandem repeats (VNTRs) in large-scale sequencing studies (Trost et al., 2020) and epigenetic mechanisms, such as DNA methylation and mRNA expression. Understanding the underlying biological mechanisms of OCD will hopefully lead to the introduction of new treatment strategies and individually tailored interventions (Zai et al., 2019). Exploring associations between genetics and psychological treatment outcome will also contribute to the development of personalized medicine in OCD (Lester & Eley, 2013).
